# What makes a good mother? Two decades of research reflecting social norms of motherhood

**DOI:** 10.1111/jftr.12488

**Published:** 2022-11-22

**Authors:** Eva‐Maria Schmidt, Fabienne Décieux, Ulrike Zartler, Christine Schnor

**Affiliations:** ^1^ Department of Sociology and Austrian Institute for Family Research University of Vienna Vienna Austria; ^2^ Department of Sociology University of Vienna Vienna Austria; ^3^ Institute of Sociology, Department for the Theory of Society and Social Analyses Johannes Kepler University Linz Linz Austria; ^4^ Centre de recherche en démographie – Centre for Demographic Research Université catholique de Louvain Louvain‐la‐Neuve Belgium

**Keywords:** individualization, motherhood, mothering, neoliberalism, practices, scoping review, social norms, WEIRD countries

## Abstract

Over the past two decades, scholars have investigated a multitude of different aspects of motherhood. This article provides a scoping review of research published from 2001 to 2021, covering 115 Social Science Citation Index‐referenced papers from WEIRD countries, with the aim of reconstructing social norms around motherhood and mothers' responses to them. The analysis is theoretically based on normological and praxeological concepts. The findings reveal five contemporary norms of motherhood that reflect both stability and increasing differentiation, and are related to five types of mothers: the norms of being attentive to the child (present mother), of securing the child's successful development (future‐oriented mother), of integrating employment into mothering (working mother), of being in control (public mother), and of being contented (happy mother). Relying on an intersectional lens, we analyze mothers' heterogeneous responses to these norms of motherhood, and examine how neoliberal demands build on and perpetuate inequalities.

For most of the preceding decades, family life in Western industrialized countries was structured by clear norms of mothers being primarily responsible for performing unpaid care and housework. With emerging neoliberal economic, political, and cultural transformations like free markets, de‐regulated and commodified social services, the demands on mothers changed, as women with children were increasingly encouraged or required to participate in paid work. Even though the societal roles of mothers and fathers have become blurred as a result of this shift (Bianchi & Milkie, 2010; Connell, 2009; Knight & Brinton, 2017; Sullivan, 2021), the division of labor and responsibilities in families has remained gendered (Grunow & Evertsson, 2019; Schmidt, Rieder, & Zartler, 2019; Schmidt, Zartler, & Vogl, 2019; Valiquette‐Tessier et al., 2019). Mothers have continued to be regarded as “good” when they invest time and energy in caring for their children (Ennis, 2014; Hays, 1996). This constellation of norms has consolidated both neoliberal tendencies and intersectional power structures.

The scholarly understanding of the contemporary norms around motherhood, and of how these norms shape mothering in the 21st century, is rather fragmented. Over the past decades, researchers have instead examined and provided several systematic reviews on parenting in general (e.g., Grunow & Evertsson, 2019; Nomaguchi & Milkie, 2020; Valiquette‐Tessier et al., 2019), or on fathers' involvement and fathering (e.g., Doucet & Lee, 2014; Ewald et al., 2020; Koslowski, 2011). The most recent systematic review of research on mothering and motherhood dates back to the beginning of the millennium, and focused on North American scholarship (Arendell, 2000). The literature reviews on this topic that have appeared since then have examined mothers' experiences and practices only in specific contexts or groups of mothers, such as single mothers (Robinson et al., 2018), mothers of children with disabilities (van Wyk & Leech, 2016), lesbian mothers (Biblarz & Savci, 2010; Bos & Gartrell, 2020), or mothers with violent partners (Hardesty & Ogolsky, 2020). An explicit and comprehensive review on mothering or on the social norms of motherhood is still lacking.

Against this background, an examination of the social norms of motherhood and of mothers' responses to them is highly relevant. Therefore, in this review, we seek to answer the following questions: Which social norms around motherhood are reflected in the research findings on mothering over the past 20 years in Western, educated, industrialized, rich, and democratic (WEIRD) countries? How have mothers responded to these norms?

This review addresses these questions by combining theories on social norms with practice theories, and analyzes the resulting challenges mothers face from a critical intersectional perspective. We approach these issues by relying on an understanding of motherhood defined as social norms and as implicit rules of endorsed and expected behavior and collectively shared systems of meaning. Individuals' concrete practices are shaped by norms without legal force. In line with practice theory, we define mothering practices as various ways of doing, thinking, or feeling; and as distinct from but linked to the social grammar of norms, and to wider systems of meanings and cultural scripts (Bicchieri, 2006, 2017; Daly, 2016; Finch, 2007; Morris et al., 2015). Mothers, the practices of mothering, and the social norms of motherhood are conceptualized as distinguishable but interrelated.

Using a qualitative procedure, we have systematically scoped the literature on mothering and motherhood from 2001 to 2021 with the aim of reconstructing social norms around motherhood. This review, which is intended to be a starting point of reference, covers internationally published and Social Science Citation Index (SSCI)‐referenced research articles on WEIRD countries (Henrich et al., 2010a, 2010b). Within this scope and in line with additional inclusion and exclusion criteria, we identified 115 scholarly articles that addressed mothering practices or mothers' experiences, or that investigated norms related to motherhood. Our findings indicate continuities and changes in the norms and an increasing diversity in mothers' responses. This growing heterogeneity entails a considerable potential for norm change. However, the tensions resulting from intensified neoliberal demands are not resolved on a normative level and continue to reinforce patriarchal and intersectional power structures. Finally, we discuss future directions for research and reviews on motherhood.

## SETTING THE CONTEXT: MOTHERHOOD IN WEIRD COUNTRIES

At least since the turn of the 21st century, countries around the world, but particularly societies defined as Western, educated, industrialized, rich, and democratic (Henrich et al., 2010a, 2010b), have been characterized by an increasing dedication to neoliberalism, with the ideal citizen being constructed as an active, self‐responsible, and self‐optimizing subject and an adult worker (Esping‐Andersen et al., 2002; Joecks, 2021; Lewis, 2001; Lightman & Kevins, 2021). Corresponding with these neoliberal demands, mothering has been framed as a highly individualized performance. Though the extent of policy support for (working) mothers varies across these countries, mothers are seen as individually responsible for their choices, and for the consequences of those choices (e.g., Connell, 2009; Glass et al., 2016; Meeussen & van Laar, 2018; Pedersen, 2016).

Nonetheless, across national contexts, gendered roles in family and paid work continue to exist, and parents' behavior remains gendered (Evertsson & Grunow, 2019; Few‐Demo & Allen, 2020). Despite the social changes that have been occurring in Western industrialized countries since the 1960s, the mother has continued to be the parent who is considered primarily responsible for childcare and for the well‐being of her (numerous) children, as well as for the household and for her (breadwinning) husband (Arendell, 2000; Bianchi, 2011; Bianchi & Milkie, 2010; Ehmer, 2021; Goldscheider et al., 2015; Scarborough et al., 2019; Valiquette‐Tessier et al., 2019). This assumption clearly serves to uphold neoliberal and patriarchal power structures in WEIRD societies. In response to the increase in mothers' rates of participation in education and paid work, feminists began to explicitly criticize these normative constructions, and called for more consideration of diversity and intersectional inequalities in mothers' experiences and challenges, particularly in North American scholarship (Arendell, 2000; Glenn et al., 1994; Hallstein et al., 2020).

In the European context, and especially since the turn of the new millennium, parenthood in general has increasingly been normatively constructed as a responsible act (Beaujouan & Berghammer, 2019). Parents, and particularly mothers, are expected to invest time and energy in their children (Berghammer & Milkie, 2021), to sacrifice themselves by placing their offspring's well‐being ahead of their own to establish strong “attachments” to their children (Faircloth, 2014; Hamilton, 2016; Hulen, 2022), and to satisfy their needs, wishes, and desires (Ennis, 2014; Hays, 1996). At the same time, early childhood education and care has been greatly expanded across countries (Koslowski et al., 2021; OECD, 2017). Scholars have increasingly recognized and investigated the resulting diversity in mothering practices, in part because of growing cultural heterogeneity, but also because of the rising numbers of cohabiting and single parents, and reconstituted families as a result of divorce or separation. Consequently, the social norms of motherhood have been questioned and challenged, as they contradict the neoliberal demands on women to be self‐responsible adult workers, and have very different meanings for different groups of mothers. Nonetheless, these norms continue to hold sway up to today.

## THEORETICAL FRAMEWORK

Given these contextual developments, conducting a systematic review of research on mothering and an analysis of the underlying norms are crucial, and potentially fruitful, endeavors. We rely on a theoretical frame of normology (Morris et al., 2015) and define social norms as rules of behavior that individuals accept, endorse, and prefer to conform to. They are shared as often implicit standards within a particular social group or context, and shape what people consider to be “good” or desirable. As the “grammar of society” (Bicchieri, 2006), they help people to navigate their social environment, and guide or constrain their behavior and practices, even in the absence of formal or legal force (Bicchieri, 2017; Morris et al., 2015). Individuals may perceive these rules as being in line with *empirical expectations*: that is, they expect that most people in their reference network conform to a norm. At the same time, individuals tend to rely on *normative expectations*: that is, they expect that most people in their reference network believe they ought to conform to the norm. These expectations are often accompanied by the belief that engaging (or failing to engage) in certain behavior will have positive (or negative) consequences (Cialdini & Trost, 1998; Fishbein & Ajzen, 1975). Accordingly, we define motherhood as “the cultural scripts and codings and institutionalized ways” (Daly, 2016, p. 52) of mothers' attributes and practices (Bergnehr, 2016).

Linking these theories of normology with foundations from practice theory, we define mothering as a set of practices (Daly, 2016). Mothering practices of performing and displaying motherhood are linked to the family, to a wider audience (Jurczyk, 2014; Morgan, 2011, 2020), and to “wider systems of meaning” (Finch, 2007, p. 67) like social norms of motherhood or societal developments. Mothers, in this theoretical framing, are defined as the actors, and as social and embodied beings. They practice mothering, which involves actions ranging from doing to thinking, feeling, aspiring, assessing, planning, and investing in aspects of their identity (Daly, 2016).

In this review, we conceive of mothers, the practices of mothering, and the social norms of motherhood as distinguishable but closely interrelated concepts. We account for the pathways through which social norms shape practices and vice versa by integrating critical feminist and intersectional theories.

First, we recognize that social norms are potentially contradictory, dissonant, and open to change; that they are context‐specific, historically and socially variable, fluid, and diverse (Daly, 2016; Morris et al., 2015; Pfau‐Effinger, 2005; Xenitidou & Edmonds, 2014). Definitions of what practices are expected and desirable may vary greatly across historic time periods and cultural and social class locations (Collins, 1994; Frederick et al., 2019; Glenn et al., 1994). Practices are also regarded as fluid and flexible (Carroll & Yeadon‐Lee, 2021; Finch, 2007; Morgan, 2020). Hence, whereas norms can be a means of reinforcing patriarchal power structures, they are also subject to negotiation. Various practices, ranging from demonstrations of compliance and support to expressions of opposition and calls for reinterpretation, might influence and change social norms (Bicchieri, 2017; Bicchieri & Mercier, 2014).

Second, and in close alignment with the contingency of norms and behavior, we conceive of mothering as being shaped by intersecting categories that create structures of domination, power, and social inequities (Collins, 1994, 2015; Few‐Demo & Allen, 2020; Frederick et al., 2019). Some scholars and feminist theorists have questioned whether a gendered focus and separate conceptualizations of fathering and mothering might maintain gendered power relations (Doucet, 2006; Fagan et al., 2014; Schmidt, 2018; Schmidt, Rieder, & Zartler, 2019; Schmidt, Zartler, & Vogl, 2019), and have argued that despite the significant neoliberal transformations, these power hierarchies remain potent between women and men, within and between families, and still need to be considered. They have also pointed to hierarchies between individual women, as mothering and motherhood norms are shaped by intersecting realities in addition to gender, including class, race, body, age, and sexual orientation (Arendell, 2000). The social norms of motherhood might have very different meanings for different groups of mothers. They might be linked to different capabilities to perform motherhood, and elicit a range of responses in mothers (e.g., Collins, 1994; Grunow & Evertsson, 2019; Schmidt, Rieder, & Zartler, 2019; Schmidt, Zartler, & Vogl, 2019; Valiquette‐Tessier et al., 2019).

Guided by this theoretical framing, we used a deductive approach in reviewing the literature. The conceptualization of social norms as endorsed and approved practices enabled us to analyze research findings that identified forms of mothering deemed to be “good” and desirable. However, the quotation marks reflect the critical feminist lens that we placed on this valuation. We systematically considered underlying structures of hierarchies and forms of oppression when analyzing normatively expected or endorsed behavior. On a macro level, this approach allowed us to identify differences in the social norms of motherhood across countries and different groups of mothers, while still considering broader international trends.

## METHODOLOGICAL PROCEDURE

We aim to provide a thorough summary and analysis of studies on motherhood that were published over the past 20 years (2001–2021).^1^ Following the framework of a scoping review (Arksey & O'Malley, 2005), we sought to determine which social norms around motherhood were addressed, and how mothers have responded to these norms.

We aimed for including articles that are ranked in the SSCI and searched the Web of Science, limited to its categories Sociology, Social Science Interdisciplinary, Women's Studies, and Family Studies. To ensure intersubjective comprehensibility, only English texts were kept in our sample. We included papers regardless of their methodological research approach (qualitative, quantitative, or mixed‐methods). We started our database search with the keywords *mothering* OR *motherhood* OR *mother* (*n* = 267.349). As a social norm is supported by and reflected in practices that are considered acceptable and valued as positive (Bicchieri, 2006; Xenitidou & Edmonds, 2014), we expected the term *good* to capture results with reference to the social norms around motherhood. We added this attribution with an AND to each of the initial keywords and identified a total of *n* = 24,818 papers. We then systematically reviewed these papers' titles and keywords in a qualitative and cyclical manner (Thomas & Harden, 2008) and identified additional theoretically relevant keywords, including *ideology*, *practice*, *norm*, *care*, *work*, *guilt*, and *feeling*. We combined the initial search terms with these additional keywords, which reduced the number of retrieved papers to *n* = 3939.

Given that neoliberal transformations have taken place above all in Western industrialized societies, we focused on studies conducted in countries that can be characterized as Western, educated, industrialized, rich, and democratic (Henrich et al., 2010a, 2010b). Our results and conclusions mainly derive from studies conducted in the Anglosphere (US, AUS, CAN, and UK) and in European countries. However, this criterion must not be equated with the characteristics of the mothers referred to in the studies, who varied in terms of their race, age, family form, financial resources, professional status, educational level, sexual identity and orientation, migration status, and ethnicity. Ages and other characteristics of mothers' children also varied.

Based on these inclusion and exclusion criteria, and on our research question, we analyzed the abstracts of *n* = 766 papers. Our analysis was inclusive with regard to disciplines, however, we excluded some papers that did not touch on sociological theorizing when, for example, examining specific experiences of mothers (like being abused by a partner, suffering drug dependency, or depression) with a psychological or social work focus. After thoroughly analyzing these studies' results and theoretical contribution, we did not expect further saturation in our findings on the theoretical systematization of social norms of motherhood.

Finally, we identified *n* = 115 papers as appropriate for further in‐depth analysis. These were grouped along the different thematic foci on mothering, mothers, and motherhood that they touched (i.e., education, consumption, external childcare, economic issues, employment, development, leisure, single mothers, displaying and networking, feeding and food, ethnic and migrant contexts, emotions and well‐being). We analyzed this data corpus to identify the social norms of motherhood that the findings reflected, even if the studies did not explicitly investigate social norms of motherhood. First, we employed a summarizing and structuring procedure of coding the papers according to the focus of their research questions (e.g., breastfeeding practices), their main findings (e.g., differences between low‐ and high‐income mothers), and the research context (e.g., country). This enabled us to develop descriptive themes, and to allocate them to the retrieved papers. Second, we examined the main findings and conclusions of the papers. Initially, two reviewers worked independently. They compared their results and integrated them into the analytical results in a cyclical process. By considering the contexts of the findings, we developed a thematic synthesis that resulted in analytical themes; that is, interpretations that went beyond the content of the original studies (Thomas & Harden, 2008). Strongly informed by our theoretical lens, we identified practices that were found to be appreciated, taken‐for‐granted, or defined as desirable, but also practices that were unexpected, unattainable, or in need of legitimization that appeared in different groups of mothers, or that elicited different responses in specific mothers. This analytical lens enabled us to distill and reconstruct the wider system of meanings and of cultural scripts, and to identify the underlying social norms.

## FINDINGS ON CONTEMPORARY NORMS OF MOTHERHOOD

Over the past two decades, scholars have investigated various aspects of mothering, such as mothers' practices and concrete activities, or their emotional and embodied status. Researchers have also analyzed how mothers and various social actors—family members, friends, members of the public, and organizations and institutions—create, reproduce, and monitor social norms of motherhood. As accepted and expected rules of behavior (Bicchieri, 2006), they are reflected in various endorsed practices or target states of mothers, in discourses about mothers, and in the strategies and reactions of mothers. In the following, we present the five norms identified in our review that appear to be strongly intertwined. Afterward, the findings on mothers' responses reflect how mothers in various social positions simultaneously have to comply with partly conflicting norms.

### The Present Mother: Norm of Being Attentive to the Child

Mothering within this norm requires a mother to have comprehensive knowledge of her child's needs and desires, which are assumed to be best met when she is physically present and highly attentive. The best response to a child's needs is connected to her expertise and to the expert knowledge that she is expected to gather. This norm is reflected in findings on mothers' efforts to monitor their children, to recognize their cues and preferences, and to fulfill their needs (e.g., Afflerback et al., 2013; Byrt & Dempsey, 2020). It is assumed that following this norm enables mothers to provide their children with the best possible care. At the same time, mothers are required to find justifications or strategies for circumstances—like bodily or financial restrictions—that prohibit them from adhering to this norm (Brookes et al., 2016; Parker & Morrow, 2017; Wallace & Chason, 2007).

Mothers feel the need to be present and to sacrifice themselves to meet this normative expectation (Carter & Anthony, 2015). Official discourses often support the view that a mother should dedicate her body, her time, and her undivided attention to her child. Breastfeeding campaigns in the UK, for example, link secure emotional attachment to the physical attachment between the child and the mother. Although they claim to promote “attachment parenting,” these campaigns address mothers, not parents (Hamilton, 2016).

Numerous studies from different national contexts have shown that this norm of being physically and emotionally present as a mother is supported by constructs like “mother knows best” and the mother as “gendered talent” (Cowdery & Knudson‐Martin, 2005). These constructs ascribe an expert status to the mother, and reflect the idealized view that no one apart from the “knowing mother” (Davis et al., 2019) is able to provide ideal caregiving for lengthy periods of time because of the mother's unique and (assumed) natural connection to the child (Christopher, 2012; Cowdery & Knudson‐Martin, 2005; Dillaway & Paré, 2008; Guendouzi, 2006). Research from Finland showed that in parental couples, it is common for both parents to value the mother's care more highly than the father's (Eerola et al., 2021; Perälä‐Littunen, 2007). Mothers tend to delegate childcare mainly to women kin, in line with the norm that women (not men) are considered to be the best caregivers for children (Christopher, 2012), and are expected to be more present and attentive. Accordingly, skepticism among mothers toward care provided by non‐family members is based in part on the notion that a present mother provides the best care (Diabaté & Beringer, 2018; Kahu & Morgan, 2007).

### The Future‐Oriented Mother: Norm of Securing the Child's Successful Development

This norm constructs a mother as the best and the most appropriate person for securing the successful development and future of her child by ensuring the child's physical and cognitive development. First, a mother is expected to optimize her child's physical development by seeking to provide the child with a nurturing environment (Dillaway, 2006), including access to healthy, low‐risk food. For example, research conducted in the United States showed that already during pregnancy, mothers are expected to choose healthy foods (Copelton, 2007; Nash, 2015). This norm includes the expectation that mothers will breastfeed after birth. Several studies reported that breast milk is considered the healthiest and most natural option, whereas formula milk is rejected as being artificial (Carter et al., 2018; Faircloth, 2010); and that breastfeeding is elevated to an “essential and non‐negotiable natural phenomenon” by official campaigns (Brookes et al., 2016, p. 361). The norm of securing a successful physical development also holds for mothers of older children when they attempt to avoid food that lacks nutrients or potentially contains harmful substances (Afflerback et al., 2013); or to provide food that is produced in an ethically and environmentally responsible manner (Cairns & Johnston, 2018; Parker & Morrow, 2017). Mothers are expected to selflessly downgrade their own needs to use their financial and time resources to secure the best possible food for their children (Cairns et al., 2013; Parker & Morrow, 2017). This norm of mothers providing an optimal basis for their children's health can also be deduced from research on mothers' practices of providing their children with purposive, meaningful, and enriching leisure, outdoor, and sports activities; and of deciding what is optimal, acceptable, or harmful behavior in their children (Clark & Dumas, 2020; Knoester & Fields, 2020; Swanson, 2009; Trussell & Shaw, 2012).

Second, mothers are expected to provide and cultivate an ideal environment for their child's successful cognitive development. A wide range of studies examined mothers' practices of disciplining, stimulating, and educating their children; their decisions regarding their children's schools and educational pathways; or their attempts to provide their children with meaningful leisure activities (e.g., Budds et al., 2017; Liss et al., 2013). This norm is reflected in findings indicating that mothers seek to creatively stimulate and educate their babies as early as possible (Budds et al., 2017; Pugh, 2005). Studies conducted in different societal contexts and national welfare systems have reported that mothers with older children seek to adhere to this norm by striving to provide them with the best possible educational environment (e.g., Baker, 2019; Byrne, 2006; Jamal Al‐deen & Windle, 2017). It has also been shown that mothers living in disadvantaged economic circumstances and regions may attempt to follow this norm by engaging in disciplining educational strategies to protect their children from poverty in the future (Lo Cricchio et al., 2019; Randles, 2021).

### The Working Mother: Norm of Integrating Employment into Mothering

This norm assumes that mothers participate in the labor market, but seek to incorporate their work responsibilities into their motherhood. Instead of viewing participation in paid work as an essential part of mothering, mothers are expected to align their professional activities with their mothering by responsibly adjusting the two life spheres. Many studies reported an expectation that if a mother engages in paid work, her work commitments should not interfere with her family responsibilities, or reduce the time and energy she has available for her children (Leigh et al., 2012; Liss et al., 2013; Pedersen, 2012; Wainwright et al., 2011). This expectation seems to serve as an implicit rule for mothers who remain in the workforce. Continued labor market attachment, particularly full‐time employment, has been viewed as detrimental to children's well‐being (Collins, 2021; Lupton & Schmied, 2002; Moilanen et al., 2020; Sniekers & van den Brink, 2019; Wall, 2013).

One way for mothers to meet the norm of the working mother is to responsibly delegate childcare to others. Some mothers consider employment as a central aspect of “good” mothering (Christopher, 2012; Dow, 2016). However, their strategies of outsourcing childcare accordingly reflect their ultimate responsibility for their child's well‐being.

The normative views regarding mothers' employment appear to be particularly challenging, as mothers often feel judged for how they balance their employment and care responsibilities, and they may feel pressure to conform to expectations that are contradictory and conflicting (Haslam et al., 2015; O'Hagan, 2018; Rodriguez Castro et al., 2022; Sniekers & van den Brink, 2019; Swenson & Zvonkovic, 2016). For example, discourses in popular magazines and in workplaces may portray working mothers in a negative light, or encourage them to provide care at home (Johnston, 2003; Sullivan, 2015). At the same time, co‐workers and employers may view women who become mothers as less committed and career‐oriented employees (O'Hagan, 2018; Thébaud & Halcomb, 2019). If a woman continues to work after having children, regardless if full‐time or part‐time, she might be considered neither an ideal employee nor a committed mother.

### The Public Mother: Norm of Being in Control

Social norms of motherhood include the expectation that a mother has a high degree of control over her own body, her personal mothering performance, and her child. In line with this norm, a mother is expected to perform, present, and negotiate acceptable forms of motherhood in an informed, conscious, and self‐aware way in both the personal sphere with family and friends, and even more so in the public realm with strangers. Research has shown how mothers are required to control their mothering based on comprehensive knowledge of the expected and accepted behavior in various sites. The expectation that a mother must be in control is reflected in mothers' attempts to be viewed positively by outsiders (Collett, 2005), or to prove that they are “good” mothers (Byrt & Dempsey, 2020; Henze‐Pedersen & Järvinen, 2021) in various phases of motherhood (Mansvelt et al., 2017). It has, for example, been shown that during pregnancy, mothers often strive to control their body size, their cravings (Copelton, 2007; Nash, 2015), and their emotions (Staneva et al., 2017). Moreover, mothers may try to control their birth procedures (Widding & Farooqi, 2016), and to demonstrate their strength and their ability to get their body under control after giving birth (Keefe et al., 2018; Maddox et al., 2020). In their attempts to be in control, mothers often follow information they gather from experts or other mothers, or base their practices on what they believe a mother is expected to do or on what feelings a mother is expected to display.

In public spaces, this norm is reflected in mothers' attempts to control their public mothering, as they expect to be observed, surveilled, assessed, supervised, and even policed by others. These attempts may, for example, occur when mothers attend children's playgroups (Collett, 2005); when they are involved in children's leisure, outdoor, and sports activities (Clark & Dumas, 2020; Clement & Waitt, 2017; Trussell & Shaw, 2012); or when they are involved in their children's school education (Caputo, 2007). Mothers are expected to know which behaviors are endorsed in which public site, and how to adapt their mothering accordingly (Grant et al., 2018; Henze‐Pedersen & Järvinen, 2021; Ponsford, 2011). They may be rewarded for their behavior, for example, by being granted access to specific groups of like‐minded mothers (Afflerback et al., 2013). It has also been shown that in addition to controlling their own behavior, mothers may seek information about and attempt to control their children's appearance, behavior, success, and performance; as their children's outcomes also function as proof of their own success (Swanson, 2009).

### The Happy Mother: Norm of Being Contented

Mothering within this norm requires mothers to adhere to the expectation of being contented and happy with their maternal role (Baker, 2019; Bergnehr & Henriksson, 2021; Carroll & Yeadon‐Lee, 2021; Clement & Waitt, 2017; Constantinou et al., 2021; Gunderson & Barrett, 2017; Jennings & Brace‐Govan, 2014). The norm of being happy with being a mother implicitly influences women's decision to have children long before they get pregnant. The belief that a woman is expected to want to become a mother (Reed et al., 2011; Staneva et al., 2017) can lead to ambivalent feelings among childless women, ranging from desire to distress (Maher & Saugeres, 2007). After her baby is born, a mother may struggle with the imperative to happily bond with and to naturally love her child (Widding & Farooqi, 2016). The norm of being contented also relates to nurturing practices. Mothers are supposed to display happiness with their decision to breastfeed, particularly when performed in public (Boyer, 2018), even though they may experience contradictory feelings, including pain, discomfort, and exhaustion; pressure from their partners or from other family members; and feelings of inadequacy (Brookes et al., 2016; Brouwer et al., 2012).

Normative expectations of how a mother is supposed to feel may also surface in digital and anonymous public spaces. It has, for example, been found that when a mother complains or expresses hate in blog or forum posts, this may be interpreted as a sign of a personal failure, which enforces the norm that motherhood is expected to evoke feelings of happiness (Mustosmäki & Sihto, 2021). Moreover, one study found that expressions of negative feelings like anger or anxiety by mothers are only tolerated if they can be justified with other normative expectations of “good” mothers, such as the expectation that a mother will keep working on these feelings until they pass (Pedersen & Lupton, 2018).

Positive emotions have been documented in mothers, including in mothers who report that they feel successful and proud because they have “produced” successful children (e.g., Budds et al., 2017; Liss et al., 2013) or “good boys” (Swanson, 2009), or have taught their children well (Mansvelt et al., 2017). This kind of success is seen as an integral part of a mother's self‐esteem and own well‐being (Collett, 2005), and mothers themselves are expected to benefit personally from leisure and outdoor activities (Knoester & Fields, 2020; Maddox et al., 2020; Miller & Brown, 2005). A mother's resulting contentedness is, in turn, seen as an important part of her own and her family's well‐being (Lloyd et al., 2016).

## MOTHERS' RESPONSES TO SOCIAL NORMS OF MOTHERHOOD: EMOTIONS, DIFFERENCES, AND INEQUALITIES

The findings clearly indicate that mothers' emotional responses to conflicting normative expectations are predominantly negative, and may even compromise mothers' well‐being (Baker, 2019; Carroll & Yeadon‐Lee, 2021; Clement & Waitt, 2017; Colodro‐Conde et al., 2015; Constantinou et al., 2021; Forbes et al., 2020; Kroska & Elman, 2009). For example, some mothers experience embodied distress and the fear of behaving and feeling deviant already during pregnancy (Nash, 2015; Staneva et al., 2017), as they consider the norm of being a happy mother to be unobtainable. Some mothers report having overwhelming emotions of regret and guilt when they do not feel happiness and motherly love after giving birth (Miller, 2007). Mothers try to make sense of and justify their feelings toward their child (Widding & Farooqi, 2016), may struggle with the “suggestive power of motherhood” (Read et al., 2012, p. 12), and experience feelings of failure, ambivalence, and confusion when they decide not to breastfeed (Brookes et al., 2016; Brouwer et al., 2012).

One of the emotional responses most prominently described in the studies included in this review is the feeling of guilt. Research conducted in different cultural and societal contexts has shown that guilt serves as a regulating force in mothers' lives as they seek to adhere to the norm of being mainly responsible for the development, health, and well‐being of their children. This norm suggests that “good” mothers are never good enough because they can always try harder (Collins, 2021). The feeling of being morally responsible for providing children with the most stimulating environment possible can turn into feelings of guilt when mothers do not meet this expectation by subduing their own needs and making constant personal sacrifices (Budds et al., 2017; Clark & Dumas, 2020; Clement & Waitt, 2017; Constantinou et al., 2021; Diabaté & Beringer, 2018; Trussell & Shaw, 2012). There is even evidence that guilt is viewed as a “natural” (Williams et al., 2013) and adequate response for mothers who do not comply with normative expectations.

The “guilt thing” (Guendouzi, 2006) occurs particularly when a mother attempts to reconcile mothering, her child's needs, her individual needs, her family's needs, and the needs of her workplace. The norm that mothers are responsible for and accessible to their children may leave mothers feeling worried about not spending enough time with their children; about the quality of daycare; and about their children's happiness; evoking feelings of guilt, personal stress, pressure, or even shame (Collins, 2021; Forbes et al., 2020; Guendouzi, 2006; Sutherland, 2010). The emotional costs of such pressures are reported by mothers in different social positions, and appear to span the course of motherhood, including later in life (Gunderson & Barrett, 2017; Mansvelt et al., 2017). In contrast, these costs are not reported by fathers (Clark & Dumas, 2020; Clement & Waitt, 2017; Trussell & Shaw, 2012), and appear to be especially high among mothers in racialized communities (Elliott et al., 2015).

Mothers' responses to feelings of guilt are diverse. Although some mothers seek to reduce guilt by quitting their job and staying home with their children, others argue that their employment makes them more present as they spend “quality time” with their children at home (Haslam et al., 2015; Sutherland, 2010). The norm that only happy mothers can be “good” mothers provides some scope for mothers to make alternative choices and to resist prescriptions of how to be present for their children: Some mothers link being a “good” mother with engaging in paid work, arguing that they would be unhappy and frustrated if they stayed home (Gunderson & Barrett, 2017; Read et al., 2012; Williams et al., 2013).

Mothers' responses to diverging normative expectations are often constructed as a choice. In line with neoliberal demands for individual decision‐making (Carter et al., 2018; Carter & Anthony, 2015; Faircloth, 2010), a mother's choices are viewed as linked to her individual responsibility to ensure her own satisfaction, and she is expected to accept all of the consequences of her decisions (Hamilton, 2016). Findings indicate that mothers' responses are varied and constrained, may involve the use of alternative strategies, and differ depending on their race, class, family form, or educational background (Carter & Anthony, 2015; Dow, 2016; Kroska & Elman, 2009; Sutherland, 2010). Social norms may be seen as impossible to adhere to or may be adapted as can be shown with four examples:

First, feeding babies and children is strongly connected with the expectation of making an informed choice based on scientific knowledge and information provided by official campaigns (Brookes et al., 2016; Faircloth, 2010). However, the available feeding options and the permissible reasons for the choice not to breastfeed are often constrained by mothers' access to resources, time, and knowledge (Afflerback et al., 2013; Brouwer et al., 2012; Cairns et al., 2013; Cairns & Johnston, 2018; Parker & Morrow, 2017). Comparisons of mothers based on race have shown that Black mothers in the US context who have limited opportunities to breastfeed construct breastfeeding as “extraordinary” given their situation, but still valuing it as optimal for a child's well‐being (Carter & Anthony, 2015).

Second, mothers' capacity to adhere to the norm of investing in their children's successful future might be constrained by intersectional inequalities. Black mothers in the UK were found to have limited capacities to choose the best education for their children due to their less privileged position (Hamilton, 2016). Accordingly, White mothers in the UK try to ensure their children's success by selecting the best schools with the “right mix” of children of different races and classes (Byrne, 2006, p. 1009). When mothers' own educational resources are devalued in the context of migration, mothers respond to the norm of securing their children's future in specific ways (Bermúdez et al., 2014; Navarro‐Cruz et al., 2021): Migrant mothers tend to invest more in their children's education than is normatively required in their country of origin to prevent downward social mobility in their children, as has been shown for Australia (Jamal Al‐deen & Windle, 2017; Ramsay, 2016). Alternatively, migrant mothers might seek to maintain the disciplining strategies of their culture of origin, even in a more permissive context, to help their children improve their school performance and achieve upward mobility, as has been shown for Sweden (Bergnehr, 2016).

Third, mothers with scarce financial resources often have concerns about being able to control or defend their own mothering (Elliott et al., 2015; Keefe et al., 2018; Wallace & Chason, 2007), and tend to attribute “poor” parenting behaviors to themselves (Cooper, 2021). Impoverished mothers were found to be continuously occupied with practices of self‐monitoring, self‐disciplining, and of adjusting their maternal behaviors (Budds et al., 2017). It has also been reported that low‐income mothers make attempts to display a positive maternal self‐image (Budds et al., 2017; Narciso et al., 2018; Vincent et al., 2010) through, for example, their “foodwork” (Karademir‐Hazır, 2021; Maher et al., 2013; Parsons et al., 2021), their “diaperwork” (Randles, 2021), or their practices of consuming clothes (Ponsford, 2011; Waight, 2019). Single mothers face particular challenges in responding to the norm of being in control. Besides finding it difficult to effectively benefit from some of the social rights provided by welfare states (see e.g., Roman, 2019), they experience social shaming and normative devaluation (Zartler, 2014). However, some single mothers have found ways of controlling and managing their performance, and of resisting stigma by explicitly valorizing their work as mothers and speaking with pride about how they bring up their children (Carroll & Yeadon‐Lee, 2021; Leonard & Kelly, 2022).

Fourth, how a mother combines paid work and childcare is often conceptualized as a personal “choice” that nobody should comment on, based on the assumption that this choice reflects a mother's individual preferences and beliefs (Debacker, 2008; Hennekam et al., 2019; Leigh et al., 2012; Wissö, 2019). However, financial circumstances also shape mothers' agency in “choosing” how much time to invest in the labor market, and evoke alternative responses to the norm of integrating employment into mothering (Crowley, 2014; Johnston & Swanson, 2006; Lyonette et al., 2011). The strategies mothers use to balance employment and parenting take various shapes, and mothers' employment decisions and their mothering ideology appear to be cyclical (Johnston & Swanson, 2006): that is, mothers align their work responsibilities with their preferred mothering, and modify their mothering ideal based on their employment decision or economic need to work (Crowley, 2014; Johnston & Swanson, 2006; Leigh et al., 2012; Loyal et al., 2017; Walls et al., 2016). Particularly if mothers have a low income, they need to develop and defend their strategies for providing enough money for their families (Elliott & Bowen, 2018; Leigh et al., 2012; Vincent et al., 2010). These mothers tend to link their success in mothering more to their sustained involvement in the labor market, and less to their physical presence in their children's lives, as for them, the benefits of working outside of the home seem to outweigh the expected costs (Leigh et al., 2012). At the same time, a mother's agency might be extended by the financial freedom, self‐reliance, and personal fulfillment she gains from paid work or professional success, particularly if the mother is in a less powerful social position (Dow, 2016; Randles, 2021), or if the mother's job is not indispensable for her family's economic survival (Christopher, 2012; Tsouroufli, 2020).

## CONCLUSIONS: CONTINUITIES AND CHANGES IN THE NORMS OF MOTHERHOOD

The aim of this qualitative scoping review was to identify the major aspects of the social norms around motherhood reflected in the findings of 115 SSCI‐indexed scientific publications over the past 20 years, and to analyze how mothers have responded to these norms. We performed a systematic search and a comparative analysis of the scholarly debate on mothering in Western, educated, industrialized, rich, and democratic (WEIRD) societies, which also included non‐rich, non‐White, non‐normative mothers in these countries. In analyzing the research findings on mothering, we applied informed critical feminist and intersectional perspectives, combined normological with practice theoretical approaches, and identified five types of mothers and corresponding normative expectations: the *present mother* who is expected to secure the best care for her child; the *future‐oriented mother* who is expected to ensure her child's success; the *working mother* who is expected to integrate her employment into her mothering; the *public mother* who is expected to control her mothering in relation to various others based on her informed status; and the *happy mother* who is expected to be contented with her role.

In the analysis, we distinguished the concept of motherhood as reflected in social norms from the concept of mothering, that is, the concrete and manifold practices of mothers (Bicchieri, 2006, 2017; Finch, 2007; Jurczyk, 2014; Morgan, 2020). This theoretical framing enabled us to uncover continuities in social norms and to show how these wider systems of meaning (Bicchieri, 2006; Daly, 2016; Finch, 2007) have become increasingly sophisticated. The different norms remain a persistent, desirable, and central reference point for mothers' practices, even if in their simultaneity they are unattainable for most, if not all, mothers, particularly for those in less privileged and less powerful positions (Connell, 2009; Glenn et al., 1994). Across national and cultural contexts, a mother's attention and caregiving is prioritized above the father's role, and above her own needs (Ennis, 2014; Hays, 1996). Related to strong neoliberal demands, mothers are expected to ensure their children's successful development, and to align their employment with these aims. The diversity in mothers' responses to social norms of motherhood is related to intersectional inequalities that constrain a mother's scope for adhering to all norms and for making choices, depending on, for example, family status, economic and educational resources, class, race or ethnic background, or her bodily abilities. Some mothers then respond with alternative practices, feelings, and strategies (Collins, 2021; Constantinou et al., 2021; Frederick et al., 2019; Guendouzi, 2006; Sullivan, 2015; Sutherland, 2010; Taylor & Wallace, 2012).

The growing heterogeneity in what is constructed as “good” mothering, as well as in mothers' diverse responses to it holds a potential for change in social norms of motherhood. When normative expectations (that most people expect mothers to conform to a norm) and empirical expectations (that most other mothers conform to a norm) become inconsistent, this widening gap expands the possibilities for slowly changing norms (Bicchieri, 2006, 2017). When enough mothers adopt alternative practices, and, for example, include breadwinning as a crucial aspect of mothering (Christopher, 2012; Dow, 2016), their behavior might weaken, extend, or change a specific norm (Bicchieri & Mercier, 2014). A shift in norms is reinforced by the increasing cultural heterogeneity (e.g., due to migration processes), which has led to a greater variety in mothers' practices, strategies, and emotions.

The potential for norm change is also facilitated by women achieving higher educational levels and social positions, which increases their income, their economic independence, and their options for outsourcing childcare. Furthermore, increasing diversity in family structures due to higher rates of divorce or separation, cohabitation, and the formation of reconstituted families may undermine longstanding role expectations for mothers, and the ways mothers deal with “moral tales” (Zartler, 2014). Another crucial driver of potential changes lies in the increasing public visibility and recognition of mothers, which has led to mothers having more opportunities for sharing knowledge and presenting various practices. Studies on public mothering in online spaces have shown that there is considerable potential for transformations, innovations, and redefinitions of mothering (Johnson, 2015; Lehto, 2020; Mackenzie, 2017, 2018; Mustosmäki & Sihto, 2021; Pedersen, 2016; Pedersen & Lupton, 2018; Rogers, 2015). As mothers also use online spaces to get information and witness different mothering, they might be empowered to change norms and legitimize alternative behavior.

The results presented in this scoping review are grounded in multiple selections and interpretations that readers have to bear in mind: first, the studies' respondents and their interpretations of their social reality; second, the researchers' selection and analysis; and, third, the selection of studies for this review and the interpretations of the respective findings by the research team. After we identified 115 SCCI‐referenced papers from WEIRD countries in a systematic search, many studies or authors that researchers (including us) would have considered important for the current state of research did not appear in this search, even though they were not systematically excluded. This refers to foundational work of particular scholars that have been given credit in the first sections of this paper, as well as to particular topics that appear to be under‐represented (e.g., studies on mothers with adult children, on digital mothering, or on motherhood in reconstituted families). Moreover, most of the included studies were published in the second half of the period we researched, which reflects the general rise of publishing in peer‐reviewed articles in indexed journals. These tendencies have generally shaped the hegemonic order of scientific knowledge, which can be seen as a significant shortcoming.

Our search strategy included a focus on “good” motherhood. Based on our awareness that the ideal of “good” mothering is crucial for maintaining gendered and intersectional power structures among mothers in WEIRD societies (Newman & Nelson, 2021), we aimed to extract findings on desired, appreciated, and expected behavior of mothers. This enabled us to identify various social norms of motherhood, even if norms were not the explicit focus of a given study. The majority of the studies pursued a qualitative approach; and although a more inclusive research perspective has emerged in recent years, most studies focused on White, middle‐class, heterosexual mothers. To enhance our understanding of contemporary motherhood and to avoid reproducing norms of “good” motherhood, future research should pursue intersectional and multiple perspective approaches. The systematic inclusion of mothers who differ in terms of class, ethnicity, race, body, age, family form, or sexuality would enable researchers to better understand the different realities of these mothers. This also holds for research conducted in WEIRD societies that seeks to capture within‐country heterogeneity among mothers and political and institutional differences across WEIRD countries. Future studies could include the scholarly debate beyond SSCI‐ranked and English‐language journals in WEIRD contexts, to also expand the normative particularities of publishing practices. Scholars should seek to avoid automatically focusing on mothers' perspectives when examining motherhood, or to equate parents with mothers, without explicitly reflecting on this tendency. Considering the perspectives of, for example, older mothers, children, grandmothers, fathers; or of other related actors like midwives, teachers, or policy‐makers; would enable researchers to assess the results on the norms of motherhood based on their relevance and their interrelation with other norms. Moreover, analyzing new kinds of data and new discourse settings, for example, in online and digital spaces, might further our understanding of contemporary motherhood.

In sum, the findings point to an intensification of the neoliberal trends of subjectification and individualization in relation to motherhood over the past 20 years in WEIRD societies: norms and mothers' practices serve the demands for maintaining women's economic productivity (Hamilton, 2016), pursuing self‐improvement (Williams et al., 2013); attaining self‐responsibility and self‐control as “good citizens” (Malatzky, 2017; Vincent et al., 2010); and producing children as self‐optimized future citizens (Lister, 2003). Mothers are expected to decide and assume responsibility for how to manage normative contradictions, how to legitimize deviant behavior or attitudes, and how to deal with the emotional and economic consequences of their decisions. Despite an increasing heterogeneity and incongruence in normative and empirical expectations toward mothers, the tensions resulting from intensified neoliberal demands are therefore not resolved on a societal level. Rather, trends of subjectification continue to reinforce patriarchal and intersectional structures of power and domination as well as inequalities between genders and between mothers in Western societies.

## Supporting information


**Appendix S1.** Supporting information.

## Footnotes

^1^
We also included papers that had been published in an online first version in 2021. Meanwhile, some of these papers have been published in a regular issue. Therefore, several references indicate 2022 as the year of publication.
