# Supplementary material for: What makes a good mother? Two decades of research reflecting social norms of motherhood
Source: J Fam Theory Rev. 2022 Nov 22;15(1):57–77. doi: 10.1111/jftr.12488 (PMC10947397; doi:10.1111/jftr.12488)
Supplement: Supplementary file 1 — Appendix S1. Supporting information. [file JFTR-15-57-s001.docx]

# Annex

**Table of selected studies**

| **Author** | **Year** | **Title** | **Country** | **Method** | **Keywords** |
| --- | --- | --- | --- | --- | --- |
| Afflerback, S.; Carter, S.; Anthony, A. K.; Grauerholz, L. | 2013 | Infant-feeding consumerism in the age of intensive mothering and risk society. | USA | Qual. | consumption; feeding/food |
| Baker, E. E. | 2019 | Motherhood, homeschooling, and mental health. | USA | Lit. | education; mother's emotions/health/well-being |
| Bergnehr, D. | 2016 | Mothering for discipline and educational success: Welfare-reliant immigrant women talk about motherhood in Sweden. | SWE | Qual. | education; migrant/ethnic/economic contexts; mother's emotions/health/well-being |
| Bergnehr, D.; Henriksson, H. W. | 2021 | Hardworking women: Representations of lone mothers in the Swedish daily press. | SWE | Qual. | single mothers |
| Bermúdez, J. M. Zak-Hunter, L. M.; Stinson, M. A.; Abrams, B. A. | 2014 | “I am not going to lose my kids to the streets”: Meanings and experiences of motherhood among Mexican-origin women. | USA | Qual. | migrant/ethnic/economic contexts |
| Boyer, K. | 2018 | The emotional resonances of breastfeeding in public: The role of strangers in breastfeeding practice. | UK | Qual. | feeding/food; mother's emotions/health/well-being |
| Brookes, G.; Harvey, K.; Mullany, L. | 2016 | ‘Off to the best start’? A multimodal critique of breast and formula feeding health promotional discourse. | UK | Qual. | feeding/food; mother's emotions/health/well-being |
| Brouwer, M. A.; Drummond, C.; Willis, E. | 2012 | Using Goffman’s theories of social interaction to reflect first-time mothers’ experiences with the social norms of infant feeding. | AUS/NZ | Qual. | feeding/food; mother's emotions/health/well-being |
| Budds, K.; Hogg, M. K.; Banister, E. N.; Dixon, M. | 2017 | Parenting agendas: An empirical study of intensive mothering and infant cognitive development. | UK | Qual. | development/leisure |
| Byrne, B. | 2006 | In search of a ‘good mix’: ‘Race’, class, gender and practices of mothering. | UK | Qual. | education; migrant/ethnic/economic contexts; mother's network/relations/audience/display |
| Byrt, A.; Dempsey, D. | 2020 | Encouraging ‘good’ motherhood: Self-tracking and the provision of support on apps for parents of premature infants. | AUS/NZ | Qual. | consumption; feeding/food; development/leisure |
| Cairns, K.; Johnston, J. | 2018 | On (not) knowing where your food comes from: Meat, mothering and ethical eating. | CAN | Qual. | consumption; feeding/food |
| Cairns, K.; Johnston, J.; MacKendrick, N. | 2013 | Feeding the ‘organic child’: Mothering through ethical consumption. | CAN | Qual. | consumption; feeding/food |
| Caputo, V. | 2007 | She’s from a ‘good family’: Performing childhood and motherhood in a Canadian private school setting. | CAN | Qual. | education |
| Carroll, N.; Yeadon-Lee, T. | 2021 | ‘I’m mum and dad in one, basically’: Doing and displaying ‘good lone motherhood’. | UK | Qual. | mother's emotions/health/well-being; single mothers |
| Carter, S. K.; Anthony, A. K. | 2015 | Good, bad, and extraordinary mothers: Infant feeding and mothering in African American mothers’ breastfeeding narratives. | USA | Qual. | feeding/food; migrant/ethnic/economic contexts |
| Carter, S. K.; Reyes-Foster, B. M.; Carter, J. S. | 2018 | “Breast is best, donor next”: Peer breastmilk sharing in contemporary western motherhood. | USA | Qual. | feeding/food |
| Christopher, K. | 2012 | Extensive mothering: Employed mothers’ constructions of the good mother. | CAN; USA | Qual. | external childcare; mothers' employment/paid work; single mothers |
| Clark, E.; Dumas, A. | 2020 | Children’s active outdoor play: ‘Good’ mothering and the organisation of children’s free time. | CAN | Qual. | development/leisure |
| Clement, S.; Waitt, G. | 2017 | Walking, mothering and care: a sensory ethnography of journeying on-foot with children in Wollongong, Australia. | AUS/NZ | Qual. | development/leisure; mother's emotions/health/well-being |
| Collett, J. L. | 2005 | What kind of mother am I? Impression management and the social construction of Motherhood. | USA | Qual. | development/leisure; mother's network/relations/audience/display |
| Collins, C. | 2021 | Is maternal guilt a cross-national experience? | GER; ITA; SWE; USA | Qual. | mother's emotions/health/well-being; mothers' employment/paid work |
| Colodro-Conde, L.; Limiñana-Gras, R. M.; Sánchez-López, M. P.; Ordoñana, J. R. | 2015 | Gender, health, and initiation of breastfeeding. | SPA | Qual. | feeding/food; mother's emotions/health/well-being |
| Constantinou, G.; Varela, S.; Buckby, B. | 2021 | Reviewing the experiences of maternal guilt - the “Motherhood Myth” influence. | AUS/NZ | Lit. | mother's emotions/health/well-being |
| Cooper, K. | 2021 | Are poor parents *poor* parents? The relationship between poverty and parenting among mothers in the UK. | UK | Quant. | migrant/ethnic/economic contexts |
| Copelton, D. A. | 2007 | “You are what you eat”: Nutritional norms, maternal deviance, and neutralization of women’s prenatal diets | USA | Qual. | feeding/food |
| Cowdery, R. S.; Knudson-Martin, C. | 2005 | The construction of motherhood: Tasks, relational connection, and gender equality. | USA | Qual. | mother's network/relations/audience/display |
| Crowley, J. E. | 2014 | Staying at home or working for pay? Attachment to modern mothering identities. | USA | Quant. | mothers' employment/paid work |
| Davis, T; Hogg, M. K.; Marshall, D.; Petersen, A.; Schneider, T. | 2019 | The knowing mother: Maternal knowledge and the reinforcement of the feminine consuming subject in magazine advertisements. | UK; AUS/NZ | Qual. | consumption |
| Debacker, M. | 2008 | Care strategies among high- and low-skilled mothers: a world of difference? | BEL | Quant. | mothers' employment/paid work |
| Diabaté, S.; Beringer, S. | 2018 | Simply the best!? The impact of cultural factors on “intensive mothering” among mothers of toddlers in Germany | GER | Quant. | external childcare; mothers' employment/paid work |
| Dillaway, H. | 2006 | Good mothers never wane: Mothering at menopause | USA | Qual. | mother's emotions/health/well-being |
| Dillaway, H.; Paré, E. | 2008 | Locating mothers: How cultural debates about stay-at-home versus working mothers define women and home. | USA | Lit. | mothers' employment/paid work |
| Dow, D. M. | 2016 | Integrated motherhood: Beyond hegemonic ideologies of motherhood. | USA | Qual. | external childcare; migrant/ethnic/economic contexts; mothers' employment/paid work |
| Eerola, P.; Närvi, J.; Terävä, J.; Repo, K. | 2021 | Negotiating parenting practices: The arguments and justifications of Finnish couples. | FIN | Qual. | mothers' employment/paid work |
| Elliott, S.; Bowen, S. | 2018 | Defending motherhood: Morality, responsibility, and double binds in feeding children. | USA | Qual. | development/leisure; feeding/food; migrant/ethnic/economic contexts |
| Elliott, S.; Powell, R.; Brenton, J. | 2015 | Being a good mom: Low-income, Black single mothers negotiate intensive mothering. | USA | Qual. | migrant/ethnic/economic contexts; single mothers |
| Faircloth, C. | 2010 | ‘What science says is best’: Parenting practices, scientific authority and maternal identity. | UK | Qual. | feeding/food |
| Forbes, L. K.; Donovan, C.; Lamar, M. R. | 2020 | Differences in intensive parenting attitudes and gender norms among U.S. mothers. | USA | Quant. | mother's emotions/health/well-being |
| Grant, A.; Mannay, D.; Marzella, R. | 2018 | ‘People try and police your behaviour’: The impact of surveillance on mothers and grandmothers’ perceptions and experiences of infant feeding. | UK | Qual. | consumption; feeding/food; mother's network/relations/audience/display |
| Guendouzi, J. | 2006 | “The guilt thing”: Balancing domestic and professional roles. | UK | Qual. | mother's emotions/health/well-being; mothers' employment/paid work |
| Gunderson, J.; Barrett, A. E. | 2017 | Emotional cost of emotional support? The association between intensive mothering and psychological well-being in midlife. | USA | Quant | mother's emotions/health/well-being |
| Hamilton, P. | 2016 | The ‘good’ attached mother: An analysis of postmaternal and postracial thinking in birth and breastfeeding policy in neoliberal Britain. | UK | Qual. | feeding/food; migrant/ethnic/economic contexts |
| Haslam, D. M.; Patrick, P.; Kirby, J. N.. | 2015 | Giving voice to working mothers: A consumer informed study to program design for working mothers. | AUS/NZ | Qual. | consumption; mothers' employment/paid work |
| Hennekam, S.; Syed, J.; Ali, F.; Dumazert, J-P. | 2019 | A multilevel perspective of the identity transition to motherhood. | NL | Qual. | mothers' employment/paid work |
| Henze-Pedersen, S; Järvinen, M. | 2021 | Displaying family at a women’s refuge. | DEN | Qual. | mother's network/relations/audience/display |
| Jamal Al-deen, T.; Windle, J. | 2017 | ‘I feel sometimes I am a bad mother’: The affective dimension of immigrant mothers’ involvement in their children’s schooling. | AUS/NZ | Qual. | education; migrant/ethnic/economic contexts |
| Jennings, L.; Brace-Govan, J.. | 2014 | Maternal visibility at the commodity frontier: Weaving love into birthday party consumption. | AUS/NZ | Qual. | consumption; mother's emotions/health/well-being |
| Johnson, S. A. | 2015 | ‘Intimate mothering publics’: Comparing face-to-face support groups and Internet use for women seeking information and advice in the transition to first-time motherhood. | AUS/NZ | Qual. | mother's network/relations/audience/display |
| Johnston, D. D. | 2003 | Invisible mothers: A content analysis of motherhood ideologies and myths in magazines. | USA | Quant. | mothers' employment/paid work |
| Johnston, D. D.; Swanson, D. H. | 2006 | Constructing the “good mother”: The experience of mothering ideologies by work status. | USA | Qual. | mothers' employment/paid work |
| Kahu, E.; Morgan, M. | 2007 | Weaving cohesive identities: New Zealand women talk as mothers and workers. | AUS/NZ | Qual. | mothers' employment/paid work |
| Karademir-Hazır, I. | 2021 | How (not) to feed young children: A class-cultural analysis of food parenting practices. | UK | Qual. | consumption; feeding/food |
| Keefe, R. H.; Brownstein-Evans, C.; | 2018 | The challenges of idealized mothering: Marginalized mothers living with postpartum | USA | Qual. | migrant/ethnic/economic contexts; mother's emotions/health/well-being |
| Knoester, C.; Fields, V. T. | 2020 | Mother-child engagement in sports and outdoor activities: Intensive mothering, purposive leisure, and implications for health and relationship closeness. | USA | Quant. | development/leisure |
| Kroska, A.; Elman, C. | 2009 | Change in attitudes about employed mothers: Exposure, interests, and gender ideology discrepancies. | USA | Quant. | mothers' employment/paid work |
| Lehto, M. | 2020 | Bad is the new good: negotiating bad motherhood in Finnish mommy blogs. | FIN | Qual. | mother's network/relations/audience/display |
| Leigh, J. P.; Pacholok, S.; Snape, T.; Gauthier, A. H. | 2012 | Trying to do more with less? Negotiating intensive mothering and financial strain in Canada. | CAN | Qual. | migrant/ethnic/economic contexts; mothers' employment/paid work |
| Leonard, M.; Kelly, G. | 2021 | Constructing the “good” mother: Pride and shame in lone mothers’ narratives of motherhood. | IRL | Qual. | migrant/ethnic/economic contexts; single mothers |
| Liss, M.; Schiffrin, H. H.; Mackintosh, V. H.; Miles-McLean, H.; Erchull, M. J. | 2013 | Development and validation of a quantitative measure of intensive parenting attitudes. | USA | Quant. | mothers' employment/paid work |
| Lloyd, K.; O’Brien, W.; Riot, C. | 2016 | Mothers with young children: Caring for the self through the physical activity space. | AUS/NZ | Qual. | development/leisure; mother's emotions/health/well-being |
| Lo Cricchio, M. G.; Lo Coco, A.; Cheah, C. S. L.; Liga, F. | 2019 | The good parent: Southern Italian mothers’ conceptualization of good parenting and parent–child relationships. | ITA | Qual. | education |
| Loyal, D.; Sutter, A.-L.; Rascle, N. | 2017 | Mothering ideology and work involvement in late pregnancy: A clustering approach. | FR | Quant | mother's emotions/health/well-being; mothers' employment/paid work |
| Lupton, D.; Schmied, V. | 2002 | “The right way of doing it all”: First-time Australian mothers’ decisions about paid employment. | AUS/NZ | Qual. | mothers' employment/paid work |
| Lyonette, C.; Kaufman, G.; Crompton, R. | 2011 | We both need to work’: Maternal employment, childcare and health care in Britain and the USA. | UK; USA | Qual. | mothers' employment/paid work |
| Mackenzie, J. | 2017 | ‘Can we have a child exchange?’ Constructing and subverting the ‘good mother’ through play in Mumsnet Talk. | UK | Qual. | mother's network/relations/audience/display |
| Mackenzie, J. | 2018 | ‘Good mums don’t, apparently, wear make-up’: Negotiating discourses of gendered parenthood in Mumsnet Talk. | UK | Qual. | mother's emotions/health/well-being; mother's network/relations/audience/display |
| Maddox, C. B.; DeLuca, J. R.; Bustad, J. J. | 2020 | Working a third shift: Physical activity and embodied motherhood. | USA | Qual. | mother's network/relations/audience/display; mother's emotions/health/well-being |
| Maher, J. M.; Saugeres, L. | 2007 | To be or not to be a mother? | AUS/NZ | Qual. | (mother's) emotions/health/well-being |
| Maher, J. M.; Wright, J.; Tanner, C. | 2013 | Responsibility and resistance: Women negotiating the nourishment of children. | AUS/NZ | Qual. | feeding/food |
| Malatzky, C. | 2017 | Australian women’s complex engagement with the yummy mummy discourse and the bodily ideals of good motherhood. | AUS/NZ | Qual. | mother's emotions/health/well-being |
| Mansvelt, J.; Breheny, M.; Stephens, C. | 2017 | Still being ‘Mother’? Consumption and identity practices for women in later life. | AUS/NZ | Qual. | consumption; mother's emotions/health/well-being |
| Miller, T. “ | 2007 | “Is this what motherhood is all about?”: Weaving experiences and discourse through transition to first-time motherhood. | UK | Qual. | mother's emotions/health/well-being |
| Miller, Y. D.; Brown, W. J. | 2005 | Determinants of active leisure for women with young children—an “ethic of care” prevails. | AUS/NZ | Qual. | development/leisure |
| Moilanen, S.; May, V.; Sevón, E.; Murtorinne-Lahtinen, M.; Laakso, M.-L. | 2020 | Displaying morally responsible motherhood: Lone mothers accounting for work during non-standard hours. | FIN | Qual. | mothers' employment/paid work; mother's network/relations/audience/display; single mothers |
| Mustosmäki, A.; Sihto, T. | 2021 | “F*** this shit” - Negotiating the boundaries of public expression of mother’s negative feelings. | FIN | Qual. | mother's emotions/health/well-being; mother's network/relations/audience/display |
| Narciso, I.; Relvas, A. P.; Ferreira, L. C.; Vieira-Santos, S.; Fernandes, M.; Santa-Bárbara, S. de; Machado, I. | 2018 | Mapping the “good mother”: Meanings and experiences in economically and socially disadvantaged contexts. | POR | Qual. | migrant/ethnic/economic contexts; mother's emotions/health/well-being |
| Nash, M. | 2015 | Indulgence versus restraint: A discussion of embodied eating practices of pregnant Australian women. | AUS/NZ | Qual. | development/leisure; mother's emotions/health/well-being |
| Navarro-Cruz, G.; Kouyoumdjian, C.; Arias, L. | 2021 | “There’s not one way to do it:”: Latina mothers’ discipline techniques. | USA | Qual. | education; development/leisure; migrant/ethnic/economic contexts |
| Newman, H.; Nelson, K. A. | 2021 | Mother needs a bigger “helper”: A critique of “wine mom” discourse as conformity to hegemonic intensive motherhood. | USA | Qual. | mother's emotions/health/well-being |
| O'Hagan, C. | 2018 | Broadening the intersectional path: Revealing organizational practices through ‘working mothers’ narratives about time. | IRL | Qual. | mothers' employment/paid work |
| Parker, B.; Morrow, O. | 2017 | Urban homesteading and intensive mothering: (Re) gendering care and environmental responsibility in Boston and Chicago. | US | Qual. | feeding/food; migrant/ethnic/economic contexts |
| Parsons, E.; Harman, V.; Cappellini, B. | 2021 | Foodwork and foodcare in hard times: Mothering, value, and values. | UK | Qual. | feeding/food; migrant/ethnic/economic contexts |
| Pedersen, D. E. | 2012 | The good mother, the good father, and the good parent: Gendered definitions of parenting. | USA | Qual. | mothers' employment/paid work |
| Pedersen, S. | 2016 | The good, the bad and the ‘good enough’ mother on the UK parenting forum Mumsnet. | UK | Qual. | mother's network/relations/audience/display |
| Pedersen, S.; Lupton, D. | 2018 | ‘What are you feeling right now?’ Communities of maternal feeling on Mumsnet. | UK | Qual. | mother's network/relations/audience/display |
| Perälä-Littunen, S. | 2007 | Gender equality or primacy of the mother? Ambivalent descriptions of good parents. | FIN | Qual./ Quant. | mother's network/relations/audience/display |
| Ponsford, R. | 2011 | Consumption, resilience and respectability amongst young mothers in Bristol. | UK | Qual. | consumption; migrant/ethnic/economic contexts; mother's network/relations/audience/display |
| Pugh, A. J. | 2005 | Selling Compromise: Toys, motherhood, and the cultural deal | USA | Quant. | consumption; development/leisure; education; mothers' employment/paid work |
| Ramsay, G. | 2016 | Black mothers, bad mothers: African refugee women and the governing of ‘good’ citizens through the Australian child welfare system. | AUS/NZ | Qual. | migrant/ethnic/economic contexts; mother's network/relations/audience/display |
| Randles, J. | 2021 | “Willing to do anything for my kids”: Inventive mothering, diapers, and the inequalities of carework. | USA | Qual. | consumption; migrant/ethnic/economic contexts |
| Read, D. M.Y.; Crockett, J.; Mason, R. | 2012 | “It was a horrible shock”: The experience of motherhood and women’s family size preferences. | AUS/NZ | Qual. | mother's emotions/health/well-being |
| Reed, S. J.; Miller, Robin L.; Valenti, M. T.; Timm, T. M. | 2011 | Good gay females and babies’ daddies: Black lesbian community norms and the acceptability of pregnancy. | USA | Qual. | migrant/ethnic/economic contexts; mother's emotions/health/well-being |
| Rodriguez Castro, L.; Brady, M.; Cook, K. | 2020 | Negotiating ‘ideal worker’ and intensive mothering ideologies: Australian mothers’ emotional geographies during their commutes. | AUS/NZ | Qual. | mothers' employment/paid work |
| Rogers, M. | 2015 | Beyond blogging: How mothers use creative non-fiction techniques in digital environments to dislodge the mask of motherhood. | AUS/NZ | Qual. | mother's network/relations/audience/display |
| Roman, C. | 2019 | Gendered and classed experiences of work–family conflict among lone mothers in Sweden. | SWE | Qual. | migrant/ethnic/economic contexts; mothers' employment/paid work; single mothers |
| Sniekers, M.; van den Brink, M. | 2019 | Navigating norms and structures: Young mothers’ pathways to economic independence. | NL | Qual. | migrant/ethnic/economic contexts; mothers' employment/paid work |
| Staneva, A. A.; Bogossian, F.; Morawska, A.; Wittkowski, A. | 2017 | “I just feel like I am broken. I am the worst pregnant woman ever”: A qualitative exploration of the “at odds” experience of women’s antenatal distress. | AUS/NZ | Qual. | mother's emotions/health/well-being |
| Sullivan, C. | 2015 | ‘Bad mum guilt’: The representation of ‘work-life balance’ in UK women’s magazines. | UK | Qual. | mother's emotions/health/well-being |
| Sutherland, J.-A. | 2010 | Mothering, guilt and shame. | USA | Lit. | migrant/ethnic/economic contexts; mother's emotions/health/well-being |
| Swanson, L. | 2009 | Soccer fields of cultural [re]production: Creating “good boys” in suburban America. | USA | Qual. | development/leisure; migrant/ethnic/economic contexts |
| Swenson, A. R.; Zvonkovic, A. M. | 2016 | Navigating mothering: A feminist analysis of frequent work travel and independence in families. | USA | Qual. | mothers' employment/paid work |
| Taylor, E. N.; Wallace, L. E. | 2012 | For shame: Feminism, breastfeeding advocacy, and maternal guilt. | USA | Lit. | feeding/food |
| Thébaud, S.; Halcomb, L. | 2019 | One step forward? Advances and setbacks on the path toward gender equality in families and work. | USA | Lit. | external childcare; mother's emotions/health/well-being; mothers' employment/paid work |
| Trussell, D. E.; Shaw, S. M. | 2012 | Organized youth sport and parenting in public and private spaces | CAN | Qual. | development/leisure; mother's network/relations/audience/display |
| Tsouroufli, M. | 2020 | Gendered and classed performances of ‘good’ mother and academic in Greece. | GRE | Qual. | migrant/ethnic/economic contexts; mothers' employment/paid work; |
| Vincent, C.; Ball, S. J.; Braun, A. | 2010 | Between the estate and the state: Struggling to be a ‘good’ mother. | UK | Qual. | migrant/ethnic/economic contexts; mother's network/relations/audience/display |
| Waight, E. | 2019 | Mother, consumer, trader: Gendering the commodification of second-hand economies since the recession. | UK | Qual. | consumption; migrant/ethnic/economic contexts |
| Wainwright, E.; Marandet, E.; Buckingham, S.; Smith, F. | 2011 | The training-to-work trajectory: pressures for and subversions to participation in the neoliberal learning market in the UK. | UK | Qual. | mothers' employment/paid work |
| Wall, G. | 2013 | ‘Putting family first’: Shifting discourses of motherhood and childhood in representations of mothers’ employment and child care. | CAN | Qual. | mothers' employment/paid work |
| Wallace, L. E.; Chason, H. | 2007 | Infant feeding in the modern world: Medicalization and the maternal body. | USA | Qual./ Quant. | feeding/food; migrant/ethnic/economic contexts |
| Walls, J. K.; Helms, H. M.; Grzywacz, J. G. | 2016 | Intensive mothering beliefs among full-time employed mothers of infants. | USA | Quant. | migrant/ethnic/economic contexts; mothers' employment/paid work; single mothers |
| Widding, U.; Farooqi, A. | 2016 | “I thought he was ugly”: Mothers of extremely premature children narrate their experiences as troubled subjects. | SWE | Qual. | mother's emotions/health/well-being |
| Williams, K.; Donaghue, N.; Kurz, T. | 2013 | “Giving guilt the flick”? An investigation of mothers’ talk about guilt in relation to infant feeding | AUS/NZ | Qual. | feeding/food |
| Wissö, T. | 2019 | What is ‘good timing’ in parenthood? Young mothers’ accounts of parenthood and its timing. | SWE | Qual. | mothers' employment/paid work |
